# A novel denitrifying methanotroph of the NC10 phylum and its microcolony

**DOI:** 10.1038/srep32241

**Published:** 2016-09-01

**Authors:** Zhanfei He, Chaoyang Cai, Jiaqi Wang, Xinhua Xu, Ping Zheng, Mike S. M. Jetten, Baolan Hu

**Affiliations:** 1 Department of Environmental Engineering, Zhejiang University, Hangzhou, China; 2 Department of Microbiology, Institute for Water and Wetland Research, Radboud University Nijmegen, Nijmegen, The Netherlands

## Abstract

The NC10 phylum is a candidate phylum of prokaryotes and is considered important in biogeochemical cycles and evolutionary history. NC10 members are as-yet-uncultured and are difficult to enrich, and our knowledge regarding this phylum is largely limited to the first species ‘*Candidatus* Methylomirabilis oxyfera’ (*M. oxyfera*). Here, we enriched NC10 members from paddy soil and obtained a novel species of the NC10 phylum that mediates the anaerobic oxidation of methane (AOM) coupled to nitrite reduction. By comparing the new 16S rRNA gene sequences with those already in the database, this new species was found to be widely distributed in various habitats in China. Therefore, we tentatively named it ‘*Candidatus* Methylomirabilis sinica’ (*M. sinica*). Cells of *M. sinica* are roughly coccus-shaped (0.7–1.2 μm), distinct from *M. oxyfera* (rod-shaped; 0.25–0.5 × 0.8–1.1 μm). Notably, microscopic inspections revealed that *M. sinica* grew in honeycomb-shaped microcolonies, which was the first discovery of microcolony of the NC10 phylum. This finding opens the possibility to isolate NC10 members using microcolony-dependent isolation strategies.

The NC10 phylum was first proposed by Rappe and Giovannoni^1^ based on environmental 16S rRNA gene sequences from flooded caves, and the phylum was named after the place where it was first detected (Nullarbor caves, Australia)^2^. In 2006, it was discovered that NC10 bacteria were connected with a novel bioprocess – anaerobic oxidation of methane (AOM) coupled to denitrification^3^. Subsequently, Ettwig *et al.*^4^ demonstrated that NC10 bacteria mediate the process of AOM coupled to nitrite reduction (Eq. 1), and these NC10 bacteria were physiologically active as denitrifying methanotrophs. Several enrichment cultures have indicated that group A members of the NC10 phylum perform AOM coupled to nitrite reduction^4^,^5^,^6^,^7^,^8^,^9^,^10^. Remarkably, the typical bacterium of the NC10 phylum, ‘*Candidatus* Methylomirabilis oxyfera’ (*M. oxyfera*), utilizes oxygen produced from nitric oxide to intra-aerobically oxidize methane under anoxic conditions^11^.


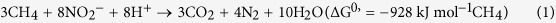


The biological process of AOM coupled to nitrite reduction links the global carbon and nitrogen cycles, and NC10 phylum bacteria may have an important role in biogeochemical processes and microbial ecology. Methane contributes approximately 20% to the greenhouse effect, and biological methane production and oxidation considerably influence the methane content in the atmosphere^12^,^13^. For this reason, the role of methane oxidation by NC10 bacteria in controlling methane emission may be important^14^,^15^,^16^,^17^,^18^,^19^. Molecular ecological analysis has showed that NC10 bacteria are abundant and have great diversity in many habitats, including freshwater systems^15^,^17^,^19^,^20^ and saline water systems^18^,^21^,^22^. However, only a few species of group A of the NC10 phylum have been enriched in the laboratory^5^,^23^,^24^. Only the first bacterium, *M. oxyfera,* has been well studied, whereas the physiology and morphology of the other NC10 bacteria remain unclear. NC10 bacteria that are distantly related to *M. oxyfera*, like group B members, are frequently detected in natural settings^15^,^17^,^19^,^20^, but the present understanding regarding the NC10 phylum is too limited to address the roles of these NC10 bacteria in the environment. Therefore, more species should be obtained in the laboratory to better understand the NC10 phylum.

To date, NC10 bacteria have not been isolated successfully, and the NC10 phylum is still a candidate division. Due to the low growth rate^3^ and possible limitation of growth factors^4^,^25^, it seems a large challenge to isolate NC10 bacteria by traditional pure culture techniques. Assuming a doubling time of 15 days, with a cell volume of 1 μm^3^, and one visible colony volume of 0.5 × 0.5 × 0.1 mm^3^, the cultivation period would be as long as one year under ideal conditions. Therefore, traditional pure culture and isolation methods are not feasible to isolate NC10 bacteria, even if these bacteria are able to grow individually on agar plates. Recently, several novel strategies were proposed to cultivate and isolate as-yet-uncultured organisms based on the microcolony formation of organisms^26^,^27^,^28^,^29^,^30^; however, no microcolonies of NC10 bacteria have been described in previous studies.

In the present work, a denitrifying methanotrophic culture was enriched from paddy soil. Multiple comparisons of the phylogeny, morphology and physiology indicated that the culture was dominated by a novel species of the NC10 phylum. The 16S rRNA and *pmoA* sequence similarity analysis of this new bacterium and 3,792 sequences retrieved from NCBI GenBank suggested this species was widely distributed in Chinese habitats. Moreover, a large number of microcolonies of NC10 bacteria were first observed in this enrichment culture.

## Results and Discussion

### Activity determination of the culture

The denitrifying methanotrophic culture was originally enriched from paddy soil with natural freshwater medium for 18 months^9^ and artificial inorganic medium^31^ for the next 42 months. In the last 6 months, the concentrations of the trace elements iron and copper (important components of the key enzymes in the central metabolism) in the medium were increased to 20 and 10 μM, respectively, to accelerate the growth of the methanotrophs^32^.

**Figure 1:**
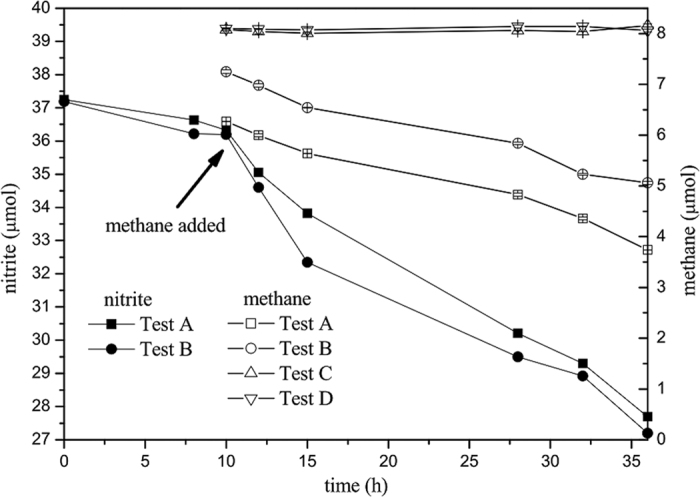
The methane oxidation and nitrite reduction activities of the culture. Tests A and B were the experimental group, whereas Tests C and D were the control group that no nitrite was supplied. In the experimental group, the initial concentration of nitrite was approximately 0.5 mM; and pure methane was added to the partial pressure of approximately 0.9 kPa after 10 hour incubation without methane, indicated by a black arrow. The methane oxidation rates were obtained directly from the best fitting of methane data, whereas the nitrite reduction rates of denitrifying methanotrophs were calculated from the nitrite reduction rate with methane (hours 10 to 36) minus the rate without methane (hours 0 to 10).

To assess the denitrifying methanotrophic activity of the culture, batch activity tests were performed, and the results are shown in Fig. 1. As expected, the methane oxidation and nitrite reduction were coupled in the culture, showing good denitrifying methanotrophic activity with a rate of 0.084 ± 0.004 μmol CH_4_ per hour, and there was no activity in the control. The nitrite reduction rate of AOM coupled to nitrite reduction process was 0.21 ± 0.01 μmol NO_2_^−^ per hour, calculated according to He *et al.*^31^. The ratio of the methane oxidation rate to the nitrite reduction rate was 3.20 ± 0.03:8, close to the stoichiometric ratio of 3:8 (Eq. 1).

### Phylogenetic analysis of the NC10 phylum

**Figure 2:**
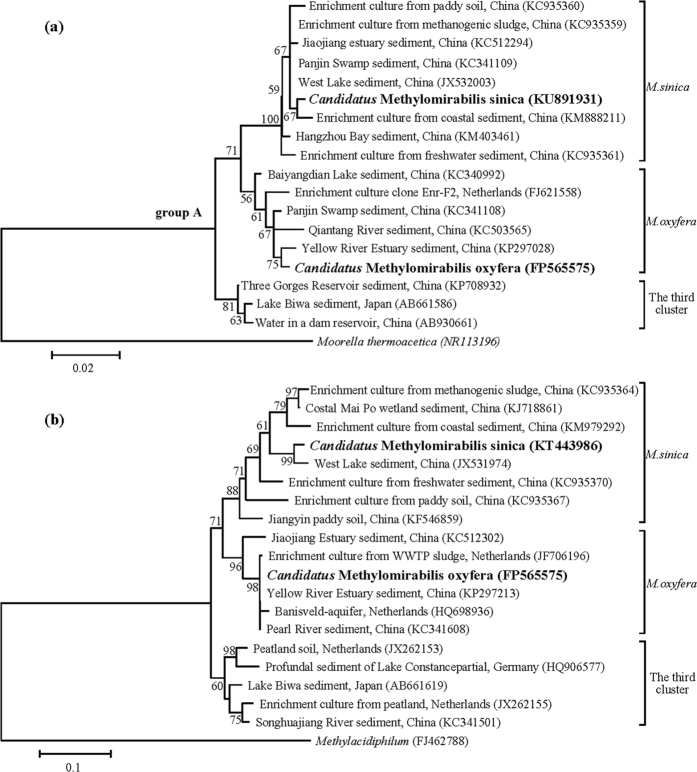
Phylogenetic trees of the 16S rRNA (**a**) and *pmoA* (**b**) gene sequences of the NC10 phylum. The trees were reconstructed using the neighbor-joining method, with *Moorella thermoacetica* (**a**) and *Methylacidiphilum* (**b**) as the out-groups. Bootstrap values (expressed as percentages of 1,000 replicates) are given at nodes. ‘*Candidatus* Methylomirabilis sinica’ are the representative gene sequences of NC10 phylum from the culture. Both 16S rRNA and *pmoA* sequences in the trees could be classified into three clusters, indicated as *M. sinica*, *M. oxyfera*, and the third cluster. A comparison of the morphology, physiology and phylogeny among the three clusters were provided in Table S3. The scale bars are 2 % (**a**) and 10 % (**b**).

Both 16S rRNA and *pmoA* gene sequences of the NC10 bacteria in the culture were phylogenetically analyzed, and the results are shown in Fig. 2. The phylogenetic analysis of the 16S rRNA genes (Fig. 2a) indicated that the representative sequence in the culture (indicated as ‘*Candidatus* Methylomirabilis sinica’, *M. sinica*) belonged to group A of the NC10 phylum but was in a distinct cluster with *M. oxyfera*. Several NC10 sequences from China were in the same cluster as the representative sequence. The sequence similarity between the representative sequence and the 16S rRNA gene sequence of *M. oxyfera* was 96.9%. The phylogenetic analysis of the *pmoA* genes (Fig. 2b) also indicated that the representative *pmoA* gene sequence (also indicated as ‘*Candidatus* Methylomirabilis sinica’) was in a distinct cluster with *M. oxyfera*. The representative *pmoA* sequence from this culture had a low sequence similarity of 85.3% to the *pmoA* sequence of *M. oxyfera*. Moreover, both 16S rRNA and *pmoA* phylogenetic trees (Fig. 2) suggested the existence of the third cluster that contained the sequences from Lake Biwa sediments^17^ and a peatland enrichment culture^23^.

**Figure 3:**
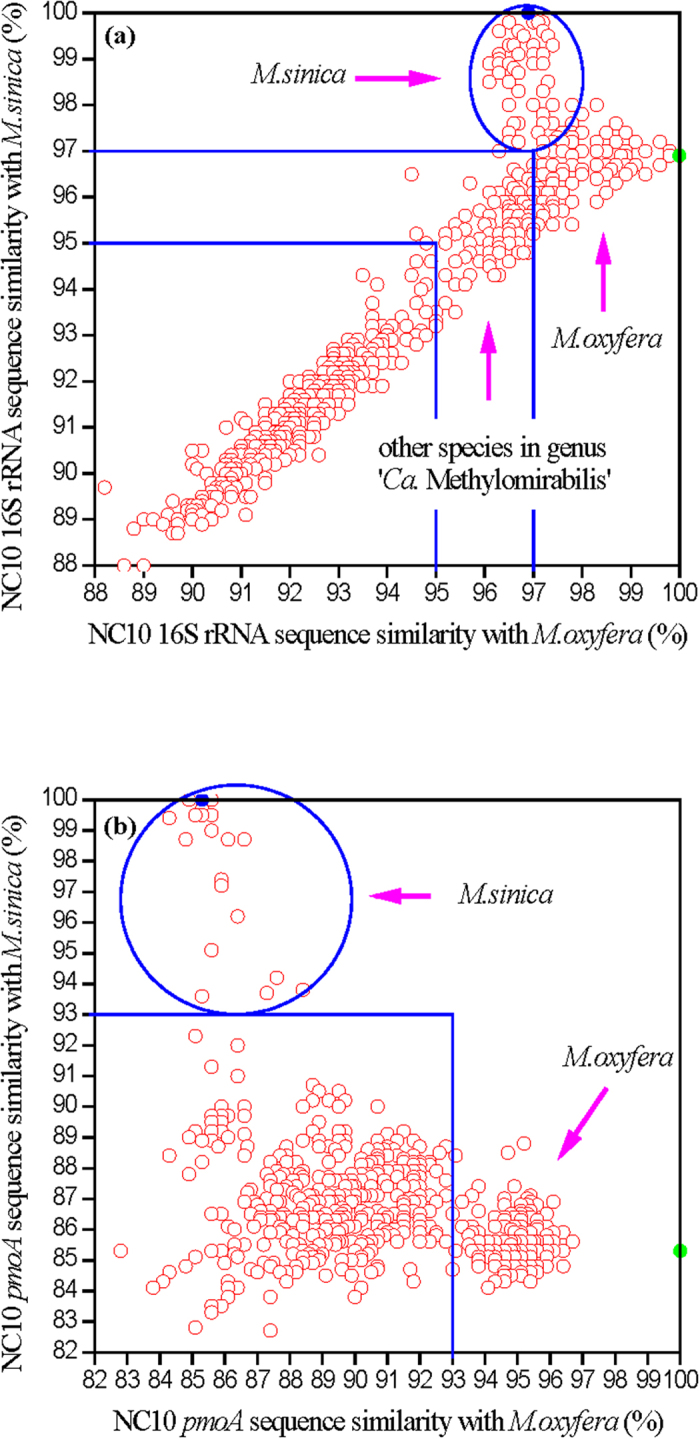
Sequence similarity analysis among the 16S rRNA (**a**) and *pmoA* (**b**) gene sequences of *M. oxyfera*, *M. sinica*, and NC10 sequences retrieved from NCBI. Every red open circle represented one sequence from NCBI; blue and green solid circles indicated *M. sinica* and *M. oxyfera*, respectively. The 16S rRNA threshold values of 97% (species) and 95% (genus) (**a**) and the *pmoA* threshold value of 93 % (species) (**b**) were shown in the figures by blue lines. The sequences belonging to *M. sinica* were marked by blue circles.

To analyze the correlations between the NC10 gene sequences obtained in this work and the sequences in the previous studies, 2,478 16S rRNA and 1,314 *pmoA* sequences of the NC10 phylum were retrieved from NCBI GenBank (date: 26-Jun-2016). The sequence similarities with *M. oxyfera* and *M. sinica* are shown in Fig. 3. 154 16S rRNA sequences not only have high similarity (>97 %) with *M. sinica* but also higher than those with *M. oxyfera* (marked in Fig. 3a); 28 *pmoA* sequences have high similarity (>93 %) with *M. sinica* (marked in Fig. 3b). According to the sequence descriptions in NCBI GenBank, these sequences were all obtained from Chinese ecosystems, including lake sediment, swamp sediment, paddy soil, forest soil, coastal sediment, estuary sediment and bay sediment.

### Microscopic observation of the culture

**Figure 4:**
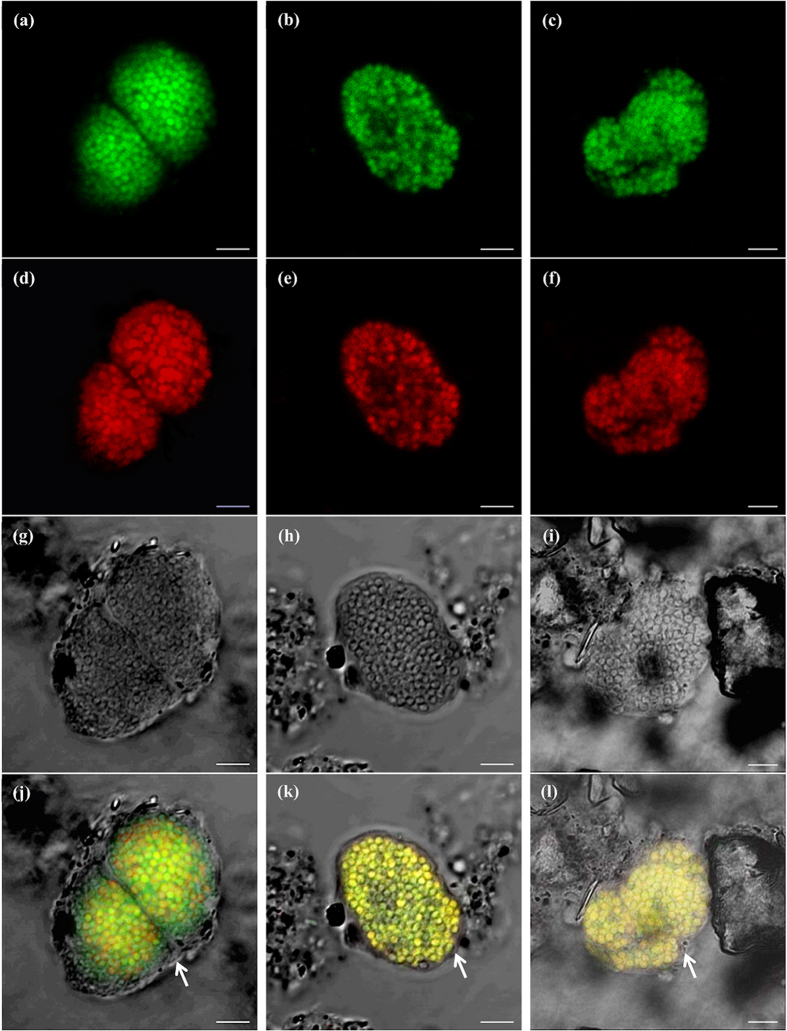
CLSM images of the microcolonies of NC10 bacteria. The cells were hybridized by a mixture of EUB I-III for most bacteria (labeled by FITC, green) (**a–c**) and a specific primer for most NC10 bacteria S-*-DBACT-1027-a-A-18 (labeled by Cy3, red) (**d–f**). The bright field images (**g–i**) were acquired from the bright field channel of CLSM. NC10 bacteria appear in yellow in the merged images (**j–l**). The images in the same column are from the identical microcolony. The dense matter surrounding the microcolonies is indicated by white arrows (**j–l**). Bar = 5 μm.

Fluorescence *in situ* hybridization (FISH) images (Fig. 4a–f) revealed that NC10 bacteria grew in a large numbers of microcolonies (clusters of the identical cells). The bright field images of the confocal laser scanning microscope (CLSM) (Fig. 4g–l) present the structure of the microcolonies clearly. The microcolonies are dense and appear in round or oval shapes with sizes of 10–30 μm. All cells of the NC10 bacteria in this culture were roughly coccus-shaped with sizes of 0.7–1.2 μm, whereas the previous NC10 bacteria (cluster *M. oxyfera* and the third cluster in Fig. 2) enriched in other laboratories were rod-shaped^5^,^6^,^23^ with a polygonal appearance under electron microscopy^33^. Close observation of the bright field images (Fig. 4g–l) suggested that the cells of our study were also polygonal (*e.g.,* pentagon, hexagon, and heptagon). Due to the polygonal shapes of the single cells and the dense structure of the microcolonies, these microcolonies resemble honeycombs, especially the microcolony in Fig. 4h. Moreover, there was some dense matter on the surfaces of the microcolonies, which is particularly clear in Fig. 4k (black line surrounded the microcolony, indicated by a white arrow), which might be important for the stability of the microcolonies.

**Figure 5:**
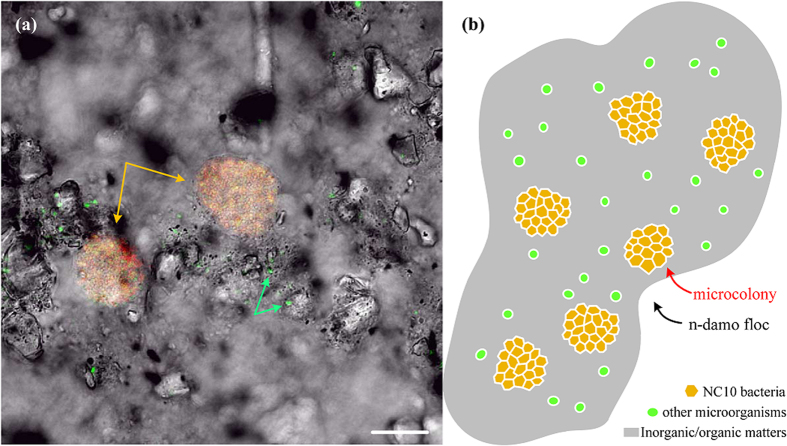
A CLSM image (**a**) and a floc conceptual model (**b**) of NC10 bacteria and other bacteria in the culture. NC10 bacteria appear in yellow (**a**) for the double hybridization of NC10 specific primer S-*-DBACT-1027-a-A-18 (red) and EUB I-III mix (green) (microcolonies are indicated by yellow arrows); and other bacteria are in green (**a**) for single hybridization of EUB I-III mix (two cells of them are indicated by green arrows). The floc conceptual model was proposed based on the CLSM image left. Scale bar is 20 μm.

All the NC10 bacteria were observed in microcolonies in the culture, and all the other organisms were detected in free cells (Fig. 5a). The microcolonies of NC10 bacteria, the free cells of other organisms and the abiotic matters together formed the flocs, and the flocs were all similar in the culture. Similar phenomena (one species of microorganism in microcolonies and the others in free cells) were also observed in other active sludge systems^30^. Based on this feature of the culture, a conceptual model of the floc was proposed that the dense microcolonies of NC10 bacteria and the free cells of other bacteria were embedded individually in the flocs, as shown in Fig. 5b. NC10 bacteria can be isolated on the basis of this feature of the culture, and the microcolonies could be selected based on the different particle sizes or settling velocities^30^,^34^.

Microcolony formation is a common behavior of microorganisms but was not described in previous studies on NC10 bacteria^5^,^6^,^8^,^23^,^24^,^35^. In environmental microbiology, microcolony formation has attracted attention due to its importance in the structure of activated sludge^36^,^37^ and the isolation of uncultured bacteria^28^,^29^,^30^. From the CLSM images, the microcolonies of NC10 bacteria are roughly spherical, dense and strong. It might be attributed to the intensive shear caused by high-rate magnetic stirring in the bioreactor. Due to poor settleability, single cells were easily withdrawn from the system with the medium exchange. Therefore, the formation of microcolony benefited NC10 bacteria “stay” in the reactor, whereas other microorganisms (single cells) were washed out when the culture was settled and the supernatant was replaced with fresh medium. Previous research indicated that extracellular DNA^37^ and other extracellular polymeric substances (EPS)^36^ were important for microcolony strength in microbial flocs and biofilms. In this work, it seemed that the dense matter (like inorganic precipitants) on the surface of the microcolonies was also important and could protect microcolonies from disintegration. The microcolony formation was long regarded as a life strategy of microorganisms under the nutrient-poor or adverse conditions^38^, and it might benefit NC10 bacteria in the competition with other microorganisms^39^, such as heterotrophic denitrifiers^24^. Moreover, the dense aggregation of cells enhanced the interactions (material, signal, gene, *etc.*) among cells^40^,^41^, and it might stimulate the growth of NC10 bacteria.

### New denitrifying methanotrophs of the NC10 phylum

The similarity of the 16S rRNA gene sequences between the *M. oxyfera* and the representative sequence in this work (positions 28 to 1,511) was 96.9%. According to the species delineation of 97% similarity and genus of 95% of the 16S rRNA gene for bacteria^42^,^43^, the representative sequence in this work represented a new species within the genus ‘*Candidatus* Methylomirabilis’. This species was first obtained in China and has only been detected in Chinese habitats, so we tentatively proposed the name ‘*Candidatus* Methylomirabilis sinica’ (*M. sinica*). The geographic distribution of this species may not be true to its name because most previous studies on NC10 bacteria in natural environments were performed in China^44^. More ecological investigations on NC10 bacteria should be performed in other countries to verify whether *M. sinica* exists in other regions. The representative sequence of the *pmoA* genes in this work (85.3% similarity to *M. oxyfera*) also showed that a new species was obtained in the culture, according to the species boundary of 93% of the *pmoA* gene for methanotrophs^45^. The activity tests (Fig. 1) demonstrated that the culture had the activity of AOM coupled to nitrite reduction. Therefore, the dominant species *M. sinica* should be a novel denitrifying methanotroph, affiliated to the genus ‘*Candidatus* Methylomirabilis’ in the NC10 phylum.

The FISH primer S-*-DBACT-1027-a-A-18 could be completely aligned to the target positions of the 16S rRNA gene sequence of *M. sinica* (Table S2), and *M. sinica* was the only NC10 bacteria in the culture (detected by 8F/1492R), which indicated that the cells hybridized by this NC10-specific primer in FISH images (Fig. 4) should be *M. sinica*. So far, only denitrifying methanotrophs in the new cluster *M. sincia* (see Fig. 2) were observed as coccus and those in other clusters are rod-shaped (Table S3). It further indicated that a new species was obtained.

### Key physiology of *M. sinica*

The important physiological parameters of *M. sinica* were determined in this study and in our previous works with the same culture. The optimal temperature and pH ranges were measured by batch experiments, and the values were 30 to 40 °C and 7.0 to 8.0, respectively^31^. *M. sinica* can grow in both freshwater^9^ and saline environments^10^. In the previous work, we obtained a halophilic NC10 culture that was also dominated by *M. sinica*^10^, and its reference sequences, KM888211 for 16S rRNA and KM979292 for *pmoA*, are shown in Fig. 2, respectively. The doubling time of *M. sinica* was approximately 25.0 days^46^, longer than that of *M. oxyfera* (1–2 weeks^11^), and the growth rate was estimated to be 0.028 ± 0.002 d^−1^ 46. The apparent substrate affinity constants for methane and nitrite were measured in this work, and they were 7.8 ± 1.2 μM and 8.9 ± 2.9 μM, respectively, similar to the results from the previous halophilic NC10 culture (9.8 ± 2.2 μM for methane and 8.7 ± 1.5 μM for nitrite^10^). The specific cell activity of *M. sinica* was approximately 0.3 fmol CH_4_ day^−1^ cell^−1^ in freshwater^47^ and 0.14 fmol CH_4_ day^−1^ cell^−1^ in saline water^10^, higher than that of *M. oxyfera* (0.09 fmol CH_4_ day^−1^ cell^−1^ 5). It may be explained by the size of the cell; *M. sinica* is significantly larger than *M. oxyfera* (0.7–1.2 × 0.7–1.2 μm *vs.* 0.25–0.5 × 0.8–1.1 μm).

### *M. sinica* bacteria in natural habitats

The phylogenetic trees (Fig. 2) and sequence similarity analyses (Fig. 3) indicated that the species *M. sinica* is widely distributed in natural environments. These *M. sinica* sequences were retrieved from freshwater systems (freshwater lake, swamp, wetland, and paddy soil)^9^,^19^ and low saline water environments (estuary, coast, and bay)^10^,^21^,^48^, but *M. sinica* sequences have not been detected in high saline water environments (such as saline lakes and deep sea)^18^,^22^. These findings suggested that *M. sinica* exists in various aquatic environments with low salinities and may be ecologically important in these ecosystems.

The existing primers for the NC10 phylum were designed based on *M. oxyfera*, and they may have bias for *M. oxyfera*. A mismatch was discovered between the sequences of the most widely used primer qP1F^5^ and *M. sinica* in this work (Table S2). This mismatch was at the last base of primer qP1F (at the 3′ end), which might influence the PCR amplification of *M. sinica* sequences. The last base of qP1F is guanine (G), but the corresponding position in the sequence of *M. sinica* is adenine (A). Therefore, the primer qP1F should be modified or redesigned to remove the PCR bias. In previous studies, the abundance and the diversity of *M. sinica* in natural habitats may have been underestimated due to this mismatch.

## Materials and Methods

### Biomass and medium

Denitrifying methanotrophs were first enriched in a previous sequencing batch reactor (SBR) with paddy soil as the initial inoculum for 18 months^9^ and were further incubated in a secondary SBR for 42 months. The characteristics of the inoculum, the configuration of the SBRs and the process of the first enrichment were previously described^9^. The biomass used in this work was harvested from the secondary SBR.

Artificial medium was prepared to feed the secondary SBR, which contained (per liter): 0.5 g KHCO_3_, 0.2 g KH_2_PO_4_, 0.3 g CaCl_2_·2H_2_O, 0.2 g MgSO_4_·7H_2_O, 0.5–1.0 g NaNO_2_, 0.2 mL alkaline trace element solution, and 0.5 mL acidic trace element solution. The alkaline trace element solution contained (per liter): 0.4 g NaOH, 0.242 g Na_2_MoO_4_·2H_2_O, and 0.05 g Na_2_WO_4_·2H_2_O. The acidic trace element solution was modified from previous literature^5^,^32^ and contained (per liter): 2.08–11.12 g FeSO_4_·7H_2_O, 0.5–5 g CuSO_4_·5H_2_O, 0.068 g ZnSO_4_·7H_2_O, 0.12 g CoCl_2_·6H_2_O, 0.5 g MnCl_2_·4H_2_O, 0.095 g NiCl_2_·6H_2_O, and 0.014 g H_3_BO_3_, and it was acidified to pH 1.0 by the addition of hydrochloric acid (HCl). The pH of the medium was adjusted to 7.2–7.4.

### Operation of the secondary SBR

A portion (approximately 0.2 L sediment) of the enrichment culture was transferred from the previous SBR into the secondary SBR. The secondary SBR consisted of 1.0 L working volume and 0.4 L headspace. The culture was incubated at 35 °C and mixed with a magnetic stirrer at 500 rpm. Every 3 days, the culture was settled for 6 hours, and 0.4 L supernatant was then replaced with an equal volume of fresh artificial medium. Subsequently, the culture was flushed with pure methane (99.99%) for approximately 10 min.

### Activity measurement

The denitrifying methanotrophic activity of the biomass was determined by batch tests. The biomass sampled from the secondary SBR was immediately washed with 10 volume nitrite- and oxygen-free medium three times. Four 62-mL serum bottles were sterilized, and each was loaded with 10 mL washed biomass and 30 mL oxygen-free medium. The four serum bottles were evenly divided into two groups: 80 μL of nitrite concentrated solution (0.5 M) was added to one group (Tests A and B), and the other (Tests C and D) did not contain nitrite (served as control group). All four serum bottles were then flushed with pure Ar (99.999%) for approximately 10 min and were sealed with grey butyl rubber stoppers. Subsequently, the biomass was incubated on a shaking table at 30 °C and 150 rpm. After 2 h pre-incubation, 0.1 mL liquid was sampled from each serum bottle. After 10 h incubation without methane, 0.2 mL pure methane (99.99%) was injected into each serum bottle. The methane in the headspace and the nitrite in the liquid were monitored after methane addition.

### DNA Extraction and PCR amplification

One milliliter of biomass was sampled and centrifuged at 7440 × *g* for 2 min. The approximately 0.25 g pellet was transferred with an aseptic stainless steel spoon to extract the total genomic DNA using the Power Soil DNA isolation kit (MoBio Laboratories Inc., USA) according to the manufacturer’s instruction manual.

The 16S rRNA gene of the bacteria was amplified using a universal primer pair 8F/1492R^49^,^50^. The PCR amplification of the NC10 phylum *pmoA* gene was performed using primer pairs A189_b/cmo682 and cmo182/cmo568, as previously described^51^. Briefly, the PCR mixtures (25 μL) contained 1 μL of template DNA, 1 μL of each primer, 9.5 μL of RNAase-free water (Takara, Japan), and 12.5 μL of Ex Taq premix (Takara, Japan) according to the manufacturer’s instruction manual. The PCR program consisted of an initial denaturation at 94 °C for 3 min, 35 cycles of denaturation at 94 °C (1 min), annealing (55 °C and 2 min for 8F/1492R; 60 °C and 1 min for A189_b/cmo682; 62 °C and 1 min for cmo182/cmo568) and extension at 72 °C (2 min for 8F/1492R; 1 min for A189_b/cmo682 and cmo182/cmo568), and a final extension at 72 °C for 10 min. The obtained PCR products were purified using agarose gel electrophoresis and Axygen PCR Cleanup kit (Axygen Scientific Inc., CA, USA). The detailed information regarding the PCR primers used above is given in Table S1.

### Cloning and sequencing

The purified PCR products were cloned in *Escherichia coli* with the pMD19-T vector (TaKaRa, Bio Inc., Shiga, Japan) according to the manufacturer’s instructions. The competent cells loaded with recombinant vectors were first incubated in SOC medium for 2 h and then grew in LB medium for 12 h at 37 °C. Ampicillin, X-Gal, and IPTG were added to the LB medium to select clones with successful ligation (blue/white screening technique)^52^. Approximately 30 positive clones from the library were sequenced by both the M13 forward and reverse primers (Invitrogen Inc., Shanghai, China). The representative sequences of the NC10 phylum were chosen using the “get.oturep” command in the Mothur v.1.36.0 program following the user instructions (http://www.mothur.org/wiki/Get.oturep) and have been deposited in the GenBank database of the National Center of Biotechnology Information (NCBI) under accession numbers KU891931 (16S rRNA) and KT443986 (*pmoA*).

### Phylogenetic analysis

Phylogenetic analyses of the NC10 sequences were performed with Mega 6.0 (Tamura *et al.* 2013), and sequences were aligned by the ClustalW algorithm. All reference sequences were retrieved from the NCBI database (http://www.ncbi.nlm.nih.gov). The neighbor-joining statistical method was used to reconstruct the phylogenetic trees with 1,000 bootstrap replicates. The sequence similarity was calculated with the ClustalW algorithm by DNAstar MegAlign software (DNAstar, USA).

### Fluorescence *in situ* hybridization (FISH)

One milliliter of biomass was sampled, centrifuged, and washed with 1 mL 1x phosphate-buffered saline (PBS; 0.01 M). Then, the samples were fixed in 0.9 mL fixation buffer (4% formaldehyde in 1x PBS) and incubated on ice for 3 h. The fixed samples were washed with 1 mL 1x PBS again and were stored in 0.5 mL 1x PBS and 0.5 mL ethanol at −20 °C^4^.

The fixed samples (10 μL) were pipetted into the wells of Teflon-coated microscope slides and dried at 46 °C in a hybridization oven. Subsequently, the samples were dehydrated in an increasing ethanol series (50, 80, and 96%) for 3 min each. After dehydration, the samples were hybridized with probes in hybridization buffer (0.9 M NaCl, 20 mM Tris/HCl pH 8.0, 0.2‰ sodium dodecyl sulfate, and 30% formamide) at 46 °C for 2 h^4^. The used oligonucleotide probes consisted of S-*-DBACT-1027-a-A-18 (5′-TCT CCA CGC TCC CTT GCG-3′) (labeled by Cy3) for NC10 bacteria (most group A and some group B)^3^ and a mixture of EUB I-III (labeled by FITC) for most bacteria^53^. After hybridization, the samples were sequentially rinsed with washing buffer (0.1 M NaCl, 20 mM Tris/HCl pH 8.0, and 5 mM EDTA pH 8.0) and Milli-Q water. Immediately, the sample was observed using a two-photon laser confocal microscope (Zeiss, LSM710 NLO, Germany). The collected micrographs were processed using the software ZEN 2012 blue edition (Carl Zeiss, Germany).

### Chemical analysis

Medium liquid samples were collected by injection syringes and passed through 0.22 μm Millipore filters. Nitrite was measured using the colorimetric method according to the APHA standard methods^54^. The gas in the headspace was extracted in triplicate to quantify the levels of methane using an Agilent 6890 gas chromatograph (Agilent, USA) equipped with a GS-CarbonPLOT capillary column (Ø 0.53 mm, 30 m length) and a flame ionization detector (FID). The temperature of the injector, oven, and detector were set at 60, 60, and 250 °C, respectively, and the carrier gas (nitrogen) flow was 2 mL min^−1^.

## Additional Information

**How to cite this article**: He, Z. *et al.* A novel denitrifying methanotroph of the NC10 phylum and its microcolony. *Sci. Rep.* **6**, 32241; doi: 10.1038/srep32241 (2016).

## Supplementary Material

Supplementary Information

## References

[b1] Rappe M. S. & Giovannoni S. J. The uncultured microbial majority. Annu Rev Microbiol 57, 369–394, doi: 10.1146/annurev.micro.57.030502.090759 (2003). 14527284

[b2] Holmes A. J. *et al.* Phylogenetic structure of unusual aquatic microbial formations in Nullarbor caves, Australia. Environmental microbiology 3, 256–264 (2001). 11359511 10.1046/j.1462-2920.2001.00187.x

[b3] Raghoebarsing A. A. *et al.* A microbial consortium couples anaerobic methane oxidation to denitrification. Nature 440, 918–921, doi: 10.1038/nature04617 (2006). 16612380

[b4] Ettwig K. F. *et al.* Denitrifying bacteria anaerobically oxidize methane in the absence of Archaea. Environmental microbiology 10, 3164–3173, doi: 10.1111/j.1462-2920.2008.01724.x (2008). 18721142

[b5] Ettwig K. F., van Alen T., van de Pas-Schoonen K. T., Jetten M. S. & Strous M. Enrichment and molecular detection of denitrifying methanotrophic bacteria of the NC10 phylum. Applied and environmental microbiology 75, 3656–3662 (2009). 19329658 10.1128/AEM.00067-09 PMC2687271

[b6] Luesken F. A. *et al.* Simultaneous nitrite-dependent anaerobic methane and ammonium oxidation processes. Applied and environmental microbiology 77, 6802–6807, doi: 10.1128/AEM.05539-11 (2011). 21841030 PMC3187098

[b7] Zhu B. *et al.* Combined anaerobic ammonium and methane oxidation for nitrogen and methane removal. Biochem Soc Trans 39, 1822–1825, doi: 10.1042/BST20110704 (2011). 22103533

[b8] Kampman C. *et al.* Enrichment of denitrifying methanotrophic bacteria for application after direct low-temperature anaerobic sewage treatment. J Hazard Mater 227–228, 164–171, doi: 10.1016/j.jhazmat.2012.05.032 (2012). 22657102

[b9] He Z. *et al.* Effect of inoculum sources on the enrichment of nitrite-dependent anaerobic methane-oxidizing bacteria. Applied microbiology and biotechnology 99, 939–946, doi: 10.1007/s00253-014-6033-8 (2015). 25186148

[b10] He Z. *et al.* Anaerobic Oxidation of Methane Coupled to Nitrite Reduction by Halophilic Marine NC10 Bacteria. Applied and environmental microbiology 81, 5538–5545, doi: 10.1128/AEM.00984-15 (2015). 26048927 PMC4510188

[b11] Ettwig K. F. *et al.* Nitrite-driven anaerobic methane oxidation by oxygenic bacteria. Nature 464, 543–548 (2010). 20336137 10.1038/nature08883

[b12] Bridgham S. D., Cadillo-Quiroz H., Keller J. K. & Zhuang Q. Methane emissions from wetlands: biogeochemical, microbial, and modeling perspectives from local to global scales. Glob Chang Biol 19, 1325–1346, doi: 10.1111/gcb.12131 (2013). 23505021

[b13] Kirschke S. *et al.* Three decades of global methane sources and sinks. Nature Geosci 6, 813–823, doi: 10.1038/ngeo1955 (2013).

[b14] Shen L. D. *et al.* Microbiology, ecology, and application of the nitrite-dependent anaerobic methane oxidation process. Frontiers in microbiology 3, 269, doi: 10.3389/fmicb.2012.00269 (2012). 22905032 PMC3408237

[b15] Hu B. L. *et al.* Evidence for nitrite-dependent anaerobic methane oxidation as a previously overlooked microbial methane sink in wetlands. Proceedings of the National Academy of Sciences of the United States of America 111, 4495–4500, doi: 10.1073/pnas.1318393111 (2014). 24616523 PMC3970540

[b16] Deutzmann J. S., Stief P., Brandes J. & Schink B. Anaerobic methane oxidation coupled to denitrification is the dominant methane sink in a deep lake. Proceedings of the National Academy of Sciences of the United States of America 111, 18273–18278, doi: 10.1073/pnas.1411617111 (2014). 25472842 PMC4280587

[b17] Kojima H. *et al.* Distribution of putative denitrifying methane oxidizing bacteria in sediment of a freshwater lake, Lake Biwa. Syst Appl Microbiol 35, 233–238, doi: 10.1016/j.syapm.2012.03.005 (2012). 22504019

[b18] Chen J., Zhou Z. C. & Gu J. D. Occurrence and diversity of nitrite-dependent anaerobic methane oxidation bacteria in the sediments of the South China Sea revealed by amplification of both 16S rRNA and *pmoA* genes. Appl. Microbiol. Biotechnol. 98, 5685–5696, doi: 10.1007/s00253-014-5733-4 (2014). 24769903

[b19] Zhu G. *et al.* Biogeographical distribution of denitrifying anaerobic methane oxidizing bacteria in Chinese wetland ecosystems. Environmental microbiology reports 7, 128–138, doi: 10.1111/1758-2229.12214 (2015). 25223900

[b20] Deutzmann J. S. & Schink B. Anaerobic oxidation of methane in sediments of Lake Constance, an oligotrophic freshwater lake. Appl. Environ. Microbiol. 77, 4429–4436, doi: 10.1128/AEM.00340-11 (2011). 21551281 PMC3127698

[b21] Shen L. D. *et al.* Molecular evidence for nitrite-dependent anaerobic methane-oxidising bacteria in the Jiaojiang Estuary of the East Sea (China). Appl. Microbiol. Biotechnol. 98, 5029–5038, doi: 10.1007/s00253-014-5556-3 (2014). 24515728

[b22] Yang J. *et al.* Co-occurrence of nitrite-dependent anaerobic methane oxidizing and anaerobic ammonia oxidizing bacteria in two Qinghai-Tibetan saline lakes. Front. Earth. Sci. 6, 383–391, doi: 10.1007/s11707-012-0336-9 (2012).

[b23] Zhu B. *et al.* Anaerobic oxidization of methane in a minerotrophic peatland: enrichment of nitrite-dependent methane-oxidizing bacteria. Applied and environmental microbiology 78, 8657–8665, doi: 10.1128/AEM.02102-12 (2012). 23042166 PMC3502929

[b24] He Z. *et al.* Nitrogen removal from wastewater by anaerobic methane-driven denitrification in a lab-scale reactor: heterotrophic denitrifiers associated with denitrifying methanotrophs. Applied microbiology and biotechnology 99, 10853–10860, doi: 10.1007/s00253-015-6939-9 (2015). 26342737

[b25] Wu M. L. *et al.* A new intra-aerobic metabolism in the nitrite-dependent anaerobic methane-oxidizing bacterium *Candidatus* ‘Methylomirabilis oxyfera’. Biochem Soc Trans 39, 243–248, doi: 10.1042/BST0390243 (2011). 21265781

[b26] Ferrari B. C., Binnerup S. J. & Gillings M. Microcolony cultivation on a soil substrate membrane system selects for previously uncultured soil bacteria. Applied and environmental microbiology 71, 8714–8720, doi: 10.1128/AEM.71.12.8714-8720.2005 (2005). 16332866 PMC1317317

[b27] Ferrari B. C., Winsley T., Gillings M. & Binnerup S. Cultivating previously uncultured soil bacteria using a soil substrate membrane system. Nat Protoc 3, 1261–1269, doi: 10.1038/nprot.2008.102 (2008). 18714294

[b28] Vartoukian S. R., Palmer R. M. & Wade W. G. Strategies for culture of ‘unculturable’ bacteria. FEMS Microbiol Lett 309, 1–7, doi: 10.1111/j.1574-6968.2010.02000.x (2010). 20487025

[b29] Pham V. H. & Kim J. Cultivation of unculturable soil bacteria. Trends Biotechnol 30, 475–484, doi: 10.1016/j.tibtech.2012.05.007 (2012). 22770837

[b30] Fujitani H., Ushiki N., Tsuneda S. & Aoi Y. Isolation of sublineage I *Nitrospira* by a novel cultivation strategy. Environmental microbiology 16, 3030–3040, doi: 10.1111/1462-2920.12248 (2014). 25312601

[b31] He Z. *et al.* The short- and long-term effects of environmental conditions on anaerobic methane oxidation coupled to nitrite reduction. Water Res 68, 554–562 (2015). 25462761 10.1016/j.watres.2014.09.055

[b32] He Z. *et al.* Improvement of the trace metal composition of medium for nitrite-dependent anaerobic methane oxidation bacteria: Iron (II) and copper (II) make a difference. Water Res 85, 235–243, doi: 10.1016/j.watres.2015.08.040 (2015). 26340061

[b33] Wu M. L. *et al.* Ultrastructure of the denitrifying methanotroph “*Candidatus* Methylomirabilis oxyfera,” a novel polygon-shaped bacterium. J Bacteriol 194, 284–291, doi: 10.1128/JB.05816-11 (2012). 22020652 PMC3256638

[b34] Zengler K. *et al.* Nonlinear partial differential equations and applications: Cultivating the uncultured. Proceedings of the National Academy of Sciences 99, 15681–15686, doi: 10.1073/pnas.252630999 (2002). PMC137776 12438682

[b35] Hu S., Zeng R. J., Keller J., Lant P. A. & Yuan Z. Effect of nitrate and nitrite on the selection of microorganisms in the denitrifying anaerobic methane oxidation process. Environmental microbiology reports 3, 315–319, doi: 10.1111/j.1758-2229.2010.00227.x (2011). 23761277

[b36] Wang B. B. *et al.* A new classification paradigm of extracellular polymeric substances (EPS) in activated sludge: separation and characterization of exopolymers between floc level and microcolony level. Water Res 64, 53–60, doi: 10.1016/j.watres.2014.07.003 (2014). 25043794

[b37] Dominiak D. M., Nielsen J. L. & Nielsen P. H. Extracellular DNA is abundant and important for microcolony strength in mixed microbial biofilms. Environmental microbiology 13, 710–721, doi: 10.1111/j.1462-2920.2010.02375.x (2011). 21118344

[b38] Tian R. M. *et al.* Effect of copper treatment on the composition and function of the bacterial community in the sponge Haliclona cymaeformis. MBio 5, e01980, doi: 10.1128/mBio.01980-14 (2014). 25370493 PMC4222105

[b39] Rao D., Webb J. S. & Kjelleberg S. Competitive interactions in mixed-species biofilms containing the marine bacterium *Pseudoalteromonas tunicata*. Applied and environmental microbiology 71, 1729–1736, doi: 10.1128/AEM.71.4.1729-1736.2005 (2005). 15811995 PMC1082554

[b40] Byun C. K. *et al.* Productive chemical interaction between a bacterial microcolony couple is enhanced by periodic relocation. Journal of the American Chemical Society 135, 2242–2247, doi: 10.1021/ja3094923 (2013). 23316688

[b41] McLean R. J. & Kakirde K. S. Enhancing metagenomics investigations of microbial interactions with biofilm technology. International journal of molecular sciences 14, 22246–22257, doi: 10.3390/ijms141122246 (2013). 24284397 PMC3856063

[b42] Konstantinidis K. T. & Tiedje J. M. Genomic insights that advance the species definition for prokaryotes. Proceedings of the National Academy of Sciences of the United States of America 102, 2567–2572, doi: 10.1073/pnas.0409727102 (2005). 15701695 PMC549018

[b43] Kim M., Oh H. S., Park S. C. & Chun J. Towards a taxonomic coherence between average nucleotide identity and 16S rRNA gene sequence similarity for species demarcation of prokaryotes. Int J Syst Evol Microbiol 64, 346–351, doi: 10.1099/ijs.0.059774-0 (2014). 24505072

[b44] Shen L. D., Wu H. S. & Gao Z. Q. Distribution and environmental significance of nitrite-dependent anaerobic methane-oxidising bacteria in natural ecosystems. Applied microbiology and biotechnology 99, 133–142, doi: 10.1007/s00253-014-6200-y (2015). 25398284

[b45] Luke C. & Frenzel P. Potential of *pmoA* amplicon pyrosequencing for methanotroph diversity studies. Applied and environmental microbiology 77, 6305–6309, doi: 10.1128/AEM.05355-11 (2011). 21764977 PMC3165393

[b46] He Z. *et al.* Modeling a nitrite-dependent anaerobic methane oxidation process: parameters identification and model evaluation. Bioresour Technol 147, 315–320 (2013). 23994967 10.1016/j.biortech.2013.08.001

[b47] Hu B. *et al.* Cultivation of nitrite-dependent anaerobic methane-oxidizing bacteria: impact of reactor configuration. Applied microbiology and biotechnology 98, 7983–7991, doi: 10.1007/s00253-014-5835-z (2014). 24880628

[b48] Chen J., Zhou Z. & Gu J. D. Complex community of nitrite-dependent anaerobic methane oxidation bacteria in coastal sediments of the Mai Po wetland by PCR amplification of both 16S rRNA and *pmoA* genes. Applied microbiology and biotechnology 99, 1463–1473, doi: 10.1007/s00253-014-6051-6 (2015). 25219532

[b49] Edwards U., Rogall T., Blöcker H., Emde M. & Böttger E. C. Isolation and direct complete nucleotide determination of entire genes. Characterization of a gene coding for 16S ribosomal RNA. Nucleic Acids Research 17, 7843–7853 (1989). 2798131 10.1093/nar/17.19.7843 PMC334891

[b50] Kane M. D., Poulsen L. K. & Stahl D. A. Monitoring the enrichment and isolation of sulfate-reducing bacteria by using oligonucleotide hybridization probes designed from environmentally derived 16S rRNA sequences. Applied and environmental microbiology 59, 682–686 (1993). 7683181 10.1128/aem.59.3.682-686.1993 PMC202174

[b51] Luesken F. A. *et al.* *pmoA* Primers for detection of anaerobic methanotrophs. Applied and environmental microbiology 77, 3877–3880, doi: 10.1128/AEM.02960-10 (2011). 21460105 PMC3127593

[b52] Emonet S. F. *et al.* Long PCR Product Sequencing (LoPPS): a shotgun-based approach to sequence long PCR products. Nat Protoc 2, 340–346, doi: 10.1038/nprot.2006.453 (2007). 17406595

[b53] Daims H., Brühl A., Amann R., Schleifer K. & Wagner M. The Domain-specific Probe EUB338 is Insufficient for the Detection of all Bacteria: Development and Evaluation of a more Comprehensive Probe Set. Syst Appl Microbiol 22, 434–444 (1999). 10553296 10.1016/S0723-2020(99)80053-8

[b54] APHA, AWWA & WEF. Standard Methods for the Examination of Water and Wastewater. (American Public Health Association, 2005).

