# Supplementary material for: A novel denitrifying methanotroph of the NC10 phylum and its microcolony
Source: Sci Rep. 2016 Sep 1;6:32241. doi: 10.1038/srep32241 (PMC5007514; doi:10.1038/srep32241)
Supplement: Supplementary Information [file srep32241-s1.doc]

*Submitted to Scientific Reports*

Supporting information for:

**A novel denitrifying methanotroph of the NC10 phylum and its microcolony**

Zhanfei He^1^, Chaoyang Cai^1^, Jiaqi Wang^1^, Xinhua Xu^1^, Ping Zheng^1^, Mike S. M. Jetten^2^, Baolan Hu^1^*

**^1^** Department of Environmental Engineering, Zhejiang University, Hangzhou, China;

**^2^** Department of Microbiology, Institute for Water and Wetland Research, Radboud University Nijmegen, Nijmegen, The Netherlands.

* Corresponding author:

*Email address:* [*blhu@zju.edu.cn*](../../../../../3%20环境因素2014.04/2014%20中间稿/2014.08%20WR%20revise/blhu@zju.edu.cn)

*Tel: 0086 0571 88982340; Fax: 0086 0571 88982340*

Table S1. The PCR primers used in this study.

Table S2. Alignment of the widely used NC10 phylum 16S rRNA gene primers with the target sequences of *M. oxyfera* and *M. sinica*.

Table S3. Comparisons of the morphology, physiology and phylogeny among the three clusters of NC10 bacteria in Fig. 2.

Table S1. The PCR primers used in this study.

| Primers | Sequence (5’-3’) | Specificity | References |
| --- | --- | --- | --- |
| 8F | AGA GTT TGA TCC TGG CTC AG | Bacteria 16S rRNA | [1](#_ENREF_1) |
| 1492R | ACG GCT ACC TTG TTA CGA CTT | Bacteria 16S rRNA | [2](#_ENREF_2) |
| A189_b | GGN GAC TGG GAC TTY TGG | NC10 *pmoA* | [3](#_ENREF_3) |
| cmo682 | AAA YCC GGC RAA GAA CGA | NC10 *pmoA* | [3](#_ENREF_3) |
| cmo182 | TCA CGT TGA CGC CGA TCC | NC10 *pmoA* | [3](#_ENREF_3) |
| cmo568 | GCA CAT ACC CAT CCC CAT C | NC10 *pmoA* | [3](#_ENREF_3) |

**Table S2. Alignment of the widely used NC10 phylum 16S rRNA gene primers with the target sequences of *M. oxyfera* and *M. sinica*.**

| Designation | Sequence | | | | | | | | | | References |
| --- | --- | --- | --- | --- | --- | --- | --- | --- | --- | --- | --- |
| qP1F | 5’ | GGG | CTT | GAC | ATC | CCA | CGA | ACC | TG | 3’ | [4](#_ENREF_4) |
| *M. oxyfera* | 5’ | GGG | CTT | GAC | ATC | CCA | CGA | ACC | TG | 3’ | [5](#_ENREF_5) |
| *M. sinica* | 5’ | GGG | CTT | GAC | ATC | CCA | CGA | ACC | TA | 3’ | This work |
| S-*-DBACT  -1027-a-A-18 | 5’ | TCT | CCA | CGC | TCC | CTT | GCG |  |  | 3’ | [6](#_ENREF_6) |
| *M. oxyfera* | 5’ | TCT | CCA | CGC | TCC | CTT | GCG |  |  | 3’ | [5](#_ENREF_5) |
| *M. sinica* | 5’ | TCT | CCA | CGC | TCC | CTT | GCG |  |  | 3’ | This work |

**Table S3.** **Comparisons of the morphology, physiology and phylogeny among the three clusters of NC10 bacteria in Fig. 2.**

| Clusters | cluster *M. sinica* | cluster *M. oxyfera* | the third cluster |
| --- | --- | --- | --- |
| 16S rRNA gene sequence similarity to *M. oxyfera* (%) | 96.1-97.4 | 97.0-100.0 | 96.4-96.9 |
| *pmoA* gene sequence similarity to *M. oxyfera* (%) | 84.3-88.4 | 93.0-100.0 | 88.0-90.2 |
| Cell shape | coccus | rod | rod |
| Cell size (μm) | 0.7-1.2 | 0.25-0.5 × 0.8-1.1 | ~0.4 × 0.8 |
| Specific cell activity  (fmol CH_4_ day^-1^ cell^-1^) | 0.14-0.3 | 0.09 | NA |
| Doubling time (d) | ~25 | 7-14 | ~60 |
| Optimal temperature (°C) | 30-40 | 20-30 | NA |
| Optimal pH ranges | 7.0-8.0 | ~7.0 | >7.0 |
| References | [7-9](#_ENREF_7) and this work |  | [12](#_ENREF_12) |

NA: not available.

**References**

1 Edwards, U., Rogall, T., Blöcker, H., Emde, M., & Böttger, E. C. Isolation and direct complete nucleotide determination of entire genes. Characterization of a gene coding for 16S ribosomal RNA. *Nucleic Acids Research* **17**, 7843-7853 (1989).

2 Kane, M. D., Lars K. Poulsen, and David A. Stahl. Monitoring the enrichment and isolation of sulfate-reducing bacteria by using oligonucleotide hybridization probes designed from environmentally derived 16S rRNA sequences. *Applied and environmental microbiology* **59**, 682-686 (1993).

3 Luesken, F. A. *et al.* *pmoA* Primers for detection of anaerobic methanotrophs. *Applied and environmental microbiology* **77**, 3877-3880, doi:10.1128/AEM.02960-10 (2011).

4 Ettwig, K. F., van Alen, T., van de Pas-Schoonen, K. T., Jetten, M. S. & Strous, M. Enrichment and molecular detection of denitrifying methanotrophic bacteria of the NC10 phylum. *Applied and environmental microbiology* **75**, 3656-3662 (2009).

5 Ettwig, K. F. *et al.* Nitrite-driven anaerobic methane oxidation by oxygenic bacteria. *Nature* **464**, 543-548 (2010).

6 Raghoebarsing, A. A. *et al.* A microbial consortium couples anaerobic methane oxidation to denitrification. *Nature* **440**, 918-921, doi:10.1038/nature04617 (2006).

7 He, Z. *et al.* The short- and long-term effects of environmental conditions on anaerobic methane oxidation coupled to nitrite reduction. *Water Res* **68**, 554-562 (2015).

8 He, Z. *et al.* Modeling a nitrite-dependent anaerobic methane oxidation process: parameters identification and model evaluation. *Bioresour Technol* **147**, 315-320 (2013).

9 Hu, B. *et al.* Cultivation of nitrite-dependent anaerobic methane-oxidizing bacteria: impact of reactor configuration. *Applied microbiology and biotechnology* **98**, 7983-7991, doi:10.1007/s00253-014-5835-z (2014).

10 Wu, M. L. *et al.* Ultrastructure of the denitrifying methanotroph "*Candidatus* Methylomirabilis oxyfera," a novel polygon-shaped bacterium. *J Bacteriol* **194**, 284-291, doi:10.1128/JB.05816-11 (2012).

11 Kampman, C. *et al.* Effect of temperature on denitrifying methanotrophic activity of '*Candidatus* Methylomirabilis oxyfera'. *Water science and technology : a journal of the International Association on Water Pollution Research* **70**, 1683-1689, doi:10.2166/wst.2014.431 (2014).

12 Zhu, B. *et al.* Anaerobic oxidization of methane in a minerotrophic peatland: enrichment of nitrite-dependent methane-oxidizing bacteria. *Applied and environmental microbiology* **78**, 8657-8665, doi:10.1128/AEM.02102-12 (2012).
